# Birthweight, gestational age, and early school trajectory

**DOI:** 10.1186/s12889-023-15913-3

**Published:** 2023-05-31

**Authors:** Rabi Joël Gansaonré, Lynne Moore, Jean-François Kobiané, Ali Sié, Slim Haddad

**Affiliations:** 1 Département de Médecine Sociale et Préventive, Faculté de Médecine, Université Laval, Québec, QC Canada; 2 Axe Santé des Populations et Pratiques Optimales en Santé, Traumatologie–Urgence-Soins intensifs, Centre de Recherche du CHU de Québec - Université Laval (Hôpital de l’Enfant- Jésus), Université Laval, Québec, QC Canada; 3 Institut Supérieur des Sciences de la Population, Université Joseph Ki-Zerbo, Ouagadougou, Burkina Faso; 4 Centre de Recherche en Santé de Nouna, Nouna, Burkina Faso; 5 Direction de la Santé Publique, Centre Intégré Universitaire de Santé et de Services Sociaux de la Capitale-Nationale, Québec, QC Canada; 6 VITAM – Centre de Recherche en Santé Durable de l’Université Laval, Québec, QC Canada

**Keywords:** Birthweight, Gestational age, School entry, Age at school entry, Grade repetition, Dropout

## Abstract

**Background:**

Birthweight and gestational age are important factors of not only newborn health by also child development and can contribute to delayed cognitive abilities. However, no study has analyzed the association of birthweight and gestational age with school trajectory measured simultaneously by school entry, grade repetition, and school dropout. This study aims, first, to analyze the association of birthweight or gestational age with school entry, and second, to explore the relationship between birthweight or gestational age and grade repetition and school dropout among children in Ouagadougou, Burkina Faso.

**Methods:**

This study used longitudinal data from the Ouagadougou Health and Demographic Surveillance System. Our samples consisted of children born between 2008 and 2014 who were at least three years old at the beginning of the 2017–18 school year. Samples included 13,676, 3152, and 3498 children for the analysis of the school entry, grade repetition, and dropout, respectively. A discrete-time survival model was used to examine the relationship between birthweight or gestational age and school entry, grade repetition, and dropout. The association between birthweight or gestational age and age at school entry were assessed using a Poisson regression.

**Results:**

The incidence rate of school entry was 18.1 per 100 people-years. The incidence of first repetition and dropout were 12.6 and 5.9, respectively. The probability of school entry decreased by 31% (HR:0.69, 95%CI: 0.56–0.85) and 8% (HR:0.92, 95%CI: 0.85–0.99) for children weighing less than 2000 g and those weighing between 2000 and 2499 g, respectively, compared to those born with a normal weight (weight ≥ 2500 g). The age at school entry of children with a birthweight less than 2000 g and between 2000 and 2499 g was 7% (IRR: 1.07, 95%CI: 1.06–1.08) and 3% (IRR: 1.03, 95%CI: 1.00-1.06) higher than children born at a normal birthweight, respectively. Gestational age was not associated with school entry or age at school entry. Similarly, birthweight and gestational age were not associated with grade repetition or dropout.

**Conclusion:**

This study shows that low birthweight is negatively associated with school entry and age at school entry in Ouagadougou. Efforts to avoid low birthweights should be part of maternal and prenatal health care because the associated difficulties may be difficult to overcome later in the child’s life. Further longitudinal studies are needed to better understand the relationship between development at birth and school trajectory.

## Introduction

In developing countries, more than 200 million children under five years old do not reach their full potential development due to poor health and nutritional status, among other factors [1]. Birthweight is an important factor of newborn health, and low birthweight can significantly contribute to delayed child development [2, 3]. Low birthweight is determined by two mechanisms that can act separately or simultaneously: gestational age and fetal growth rate [4, 5]. There is also increasing evidence that people born preterm are at risk of a spectrum of developmental morbidities (e.g., autism spectrum, cerebral palsy) from birth to adulthood [6].

Previous studies have shown, for example, that children born at a low birthweight and preterm had higher risks of death, growth impairment, and delayed neurodevelopment compared to normal-birthweight and term-birth children [2, 6, 7]. Studies indicate an association between child development at birth and their future cognitive and behavioral development [8, 9]. Several researchers have also studied the association between birthweight or gestational age and children’s performance on standardized reading and mathematics tests, behavioral problems, and cognitive development [10–17]. These studies globally found that people with low birthweight score lower in mathematics, language, and cognitive tests compared to those with normal birthweight, although a few studies [10–12] have failed to establish a significant relationship. Similarly, premature children are more likely to have poor school performances compared to term-birth children [6, 15, 17], even if some studies did not find a significant association [14, 16].

Another set of studies focused on the association between birthweight and outcomes related to school trajectory measured by grade repetition and educational attainment [13, 18–20]. However, some studies did not find significant relationships between low birthweight and school performance and school attainment [18, 21]. Therefore, the current scientific knowledge does not give us clear picture of the possible effects of low birthweight or of preterm birth and their magnitude. Furthermore, most of the previous studies have focused he relationship of birthweight with cognitive abilities and achievement on test scores. However, to measure school-age children’s academic or test achievements, children must first enter school.

Research that studied grade repetition measured the outcome at a particular point in time, which does not necessarily reflect the complexity of children’s school trajectory. In fact, the school trajectory can be strewn with different problematic situations: (1) whether the child entered at school at the legal age (e.g., 6 years) or the entry was delayed [22]; (2) an occurrence of repetition, which will slow down the child’s progress [22, 23]; (3) and an early school dropout [22, 23]. To our knowledge, no study has yet analyzed the association of birthweight or gestational age with school trajectory measured by age at school entry, grade repetition, and school dropouts.

Our primary objective was to estimate the association of birthweight or gestational age with school entry. We hypothesize that low-birthweight (birthweight < 2500 g) or preterm-birth (gestational age < 37 weeks’ gestation) children are less likely to enter school. A secondary objective was to explore the relationship between birthweight or gestational age and grade repetition and school dropout.

## Methods

We used data from the Ouagadougou Health and Demographic Surveillance System (OHDSS). Since 2008 the OHDSS has been following more than 80,000 individuals, collecting health and demographic data in two formal and three informal neighborhoods in the periphery of the city of Ouagadougou, Burkina Faso’s capital city [24]. Inhabitants of the area are mainly migrants from rural areas. Public infrastructure (water, electricity, schools, and health centers) are only available in formal neighborhoods [24].

Families are visited every 10 months. Data are collected on vital events such as births, unions, migrations, and deaths [24]. For each birth, place of delivery, number of children born at delivery, child weight and height are registered. The OHDSS also gathers data on education, employment, and vaccination. The survey also collects data on school attendance and class attended by children of the panel. Education data is available for five school years, from 2013 to 14 to 2017–18. Data are available from the Institut Superieur des Sciences de la Population, but use requires authorization.

Ouagadougou provides an appropriate research context for analyzing the link between development at birth and schooling. The issues of access to school and development at birth have evolved significantly in the city since the beginning of the 2000s. Indeed, the Demographic and Health Survey Program estimated the proportion of low birthweight (< 2500 g) in Ouagadougou at 20.8% and 15.5% among births during the three years prior to the 2003 and 2010 surveys, respectively [25]. Between 1998 and 2010, the access rate to the first grade of the elementary school increased from 85.1–92.4% [26]. These changes could be due to decision-makers’ desire to improve children’s health and access to education. For example, the government of Burkina Faso implemented two main education policies—Plan Décennal de Développement de l’Enseignement de Base (2002–2011) and the Programme de Développement Stratégique de l’Éducation de Base (2012–2021)—aimed to increase the supply of education and reduce disparities between genders, geographical regions, and socioeconomic situations [26–28]. In 2007 the government of Burkina Faso made schooling mandatory for both boys and girls between the ages of 6 and 16 [29]. Although access to school tends to be universal in the capital, many children leave school before completing the elementary cycle as indicated by the elementary school completion rate of 69.9% in 2010 [26].

### Study design and participants

Data on child development at birth were merged with longitudinal education data using a unique identifier. Our samples consisted of children born between 2008 and 2014 who were at least three years old at the beginning of the 2017–18 school year. The first sample comprised 13,676 children. This sample allowed us to test the hypothesis that low-birthweight or preterm-birth children are less likely to enter school. The second sample consisted of 3152 children who have been in school for at least two school years. This sample allowed us to examine the relationship between birthweight or gestational age and grade repetition. To analyze the association between development at birth and dropout rates, we used a third sample of 3498 children who have already been to school (that is, some of them are no longer in school and the others are still in school). A subsample of 510 children aged 9 and over who have already been to school was considered for the analysis of the relationship between development at birth and age at school entry. We assume that from the age of 9, children will no longer enter school.

### Outcome variables

The education surveys collect information on grades attended or last grade attended and education status (goes to school, has gone to school at some point but is no longer in school, has never been to school) for each participant. We created four outcome variables. The first outcome, school entry, was coded 1 when a child entered at the first grade of elementary school. A second outcome was age at school entry, the age at which the child enters school for the first time. The third was grade repetition. It takes the value 1 if grade attended at time t + 1 is equal to grade attended at time t at any round of the survey. The fourth outcome was school dropouts. We assume that there is a dropout if education status at time t is “has gone to school.” All outcomes, except the age at school entry, were right censored.

### Exposure variables

Exposure variables were birthweight and gestational age. Birthweight has been considered binary in many previous studies in this field. However, in the present study, to take into account different levels of low birthweight, and based on previous studies [30, 31], birthweight has been categorized into three groups: ≤ 1999 g, 2000–2499 g, and ≥ 2500 g. Gestational age is defined as the number of weeks between date of last normal period and the date of birth [32]. Gestational age was calculated as the difference between the date of birth and the estimated date of the beginning of the pregnancy, which was provided by the mother. Then, it was categorized as very preterm birth (≤ 31 weeks), moderate preterm birth (32–36 weeks), and normal term birth (≥ 37 weeks). Missing data on covariates were handle by the analysis method used and described below.

### Potentially confounders or modifiers

Aside from child development variables, some characteristics of children, mothers, and households were introduced in the models as covariates because they influence education and eventually child health [3, 22, 33–35]. The children’s characteristics were defined by sex and birth rank; the mother’s characteristics were education level and age at childbirth; and household characteristics referred to place of residence and socioeconomic status. Place of residence (formal or informal) allows also to control for education supply [22]. Socioeconomic status is a proxy based on material possessions (e.g., television, refrigerator) and the highest value means of transportation owned by the household (described in detail elsewhere [36]). Principal component analysis (PCA) was used to determine factorial axis and the first factor was recoded into three categories (poor, middle class, and rich). For this analysis, the variable was coded into two categories (poor and less-poor) because of the small proportion of rich in our sample.

### Statistical analysis

A discrete-time survival model was used to examine the association between child development at birth and the probability of experiencing school entry, grade repetition, and dropout during the follow-up period. The discrete-time survival model allowed us to estimate the conditional probability that an individual will experience an event at a particular time, given that the event has not yet occurred for this individual [37]. As recommended by Allison [37], the model was fitted through logistic regression. Associations between birthweight or gestational age and age at school entry were assessed using Poisson regression [38, 39].

At first, the associations between each dependent variable and development at birth were adjusted for all covariates. A time variable that represents the interval time between two school years was included in the discrete-time model. Using maximum likelihood robust estimator for robust standard error, 95% confidence intervals were estimated. This also allowed us to handle overdispersion in the Poisson model [39].

For the estimation of the association across groups, interaction terms between covariates (child sex, mother’s education, socioeconomic status, and place of residence) and the exposure variables were introduced in the models. For all models, a full information maximum likelihood (FIML) approach was used to handle missing covariate data [38, 40]. FIML provides unbiased and efficient estimators under ignorable missing data assumptions [41]. All analysis was performed using Mplus 8.6.

For the analysis of grade repetition and dropout, a selection bias can occur if, for example, children born with low birthweight are less likely to be enrolled. To try to overcome this possible selection bias, the models were estimated by the inverse probability weighting (IPW) of selection [42]. A logistic regression was fitted to estimate the probability of being selected [43]. Child sex, mother’s education, socioeconomic status, and place of residence, birth order, child’s relationship to the household’s head, and household size were included in the model as covariates. The stabilized weight, which corresponds to the inverse of the probability of being selected, was then created [43]. Then, we estimated our final models, weighing them by the IPW of selection.

## Results

Table 1 shows descriptive statistics according to each sample. Low-birthweight (< 2500 g) and preterm-birth children (< 37 weeks) represent 12.5% and 23.7% of school entry sample (sample 1), respectively. From the grade repetition sample (sample 2), 10.4% and 27.0% of children were born at low birthweight and preterm, respectively. For the dropout sample (sample 3), 10.6% had a low birthweight and 26.8% were born before 37 weeks.

**Table 1:** Baseline characteristics of participants

Characteristics		Sample 1 School entry			Sample 2 Repetition			Sample 3 Dropout	
		N (13,676)	%		N (3152)	%		N (3498)	%
Birthweight									
≤ 1999		227	1.7		34	1.1		39	1.1
2000–2499		1384	10.1		265	8.4		302	8.7
≥ 2500		10,472	76.6		2590	82.2		2862	81.8
Missing		1593	11.6		263	8.3		295	8.4
Gestational age									
≤ 31		466	3.4		94	3.0		109	3.1
32–36		2666	19.5		734	23.3		803	23.0
≥ 37		10,059	73.6		2242	71.1		2494	71.3
Missing		485	3.5		82	2.6		92	2.6
Child’s sex									
Male		6820	49.9		1557	49.4		1718	49.1
Female		6856	50.1		1595	50.6		1780	50.9
Birth rank									
1		5621	41.1		1216	38.6		1374	39.3
2		2749	20.1		767	24.3		855	24.5
3		1833	13.4		528	16.8		571	16.3
4 or more		1668	12.2		511	16.2		550	15.7
Missing		1258	9.2		130	4.1		148	4.2
Mother’s age at child’s birth (mean (sd))		13,659^¶^	26.4 (5.8)		3146^§^	26.3 (5.9)		3492 ^ρ^	26.3 (5.9)
Mother’s education									
None		6131	44.8		1473	46.7		1635	46.7
Some education		5878	43.0		1585	50.3		1752	50.1
Missing		1667	12.2		94	3.0		111	3.2
Household socioeconomic status									
Less-poor		9809	71.7		2573	81.6		2841	81.2
poor		2528	18.5		579	18.4		655	18.7
Missing		1339	9.8		0	0.0		2	0.1
Place of residence									
Formal		4624	33.8		1075	34.1		1211	34.6
Non formal		9052	66.2		2077	65.9		2287	65.4

^¶^ Missing = 17 (0.05%) ; ^§^ Missing = 6 (0.19%) ; ^ρ^ Missing = 6 (0.17%);

The incidence rate of school entry was 18.1 per 100 people-years, while the incidence of first-grade repetition and dropout were 12.6 and 5.9 per 100 people-years, respectively (Table 2).

**Table 2:** incidence rate of school entry, grade repetition and dropout according to birthweight and gestational age

Independent variable	School entry (per 100 people-years)	Repetition (per 100 people-years)	Dropout (per 100 people-years)
Birthweight			
≤ 1999	12.6	13.3	5.4
2000–2499	16.9	14.4	6.7
≥ 2500	18.3	12.4	5.8
Gestational age			
≤ 31	17.9	14.1	7.2
32–36	19.2	11.8	5.3
≥ 37	17.7	12.7	6.1
Total	18.1	12.6	5.9

Table 3 presents the association of birthweight or gestational age with school entry, grade repetition, and dropout. Results suggest that being born with a weight less than 2000 g is associated with a 31% (HR: 0.69, 95% CI: 0.56–0.85) decrease in the probability of school entry, compared to normal-birthweight children. This probability was 0.92 (95% CI: 0.85–0.99), a decrease of 8%, for children born with weight between 2000 and 2499 g.

**Table 3:** Association of birthweight and gestational age with school entry, grade repetition, and dropout

Independent variable		School entry			Repetition			Dropout	
		HR	95% CI		HR	95% CI		HR	95% CI
Birthweight (ref. BW ≥ 2500)									
≤ 1999		0.69	0.56–0.85		1.17	0.22–2.12		0.94	0.52–1.35
2000–2499		0.92	0.85–0.99		1.39	0.89–1.88		1.43	0.44–2.42
Gestational age (ref. GA ≥ 37)									
≤ 31		1.01	0.90–1.11		0.82	0.43–1.56		0.97	0.35–1.57
32–36		1.05	1.00-1.09		1.00	0.75–1.35		0.94	0.55–1.35

HR: Hazard rate; CI: Confidence interval; BW: Birthweight; GA: Gestational age; all models were adjusted for sex, birth rank, mother’s education, mother’s age at childbirth, household socioeconomic status, and place of residence.

School entry of low-birthweight children was more likely to be delayed. In fact, the age at school entry of children with a birthweight lower than 2000 g was 1.07 (95% CI: 1.06–1.08) times higher than the age at school entry of children whose birthweight was 2500 g or more (Table 4). The age at school entry was 1.03 (95% CI: 1.00-1.06) times higher for those weighing between 2000 and 2499 g at birth (Table 4).

**Table 4:** Association of birthweight and gestational age with age at school entry

Independent variable		Age at school entry		
		IRR	95% CI	
Birthweight (ref. BW ≥ 2500)				
≤ 1999		1.07	1.06–1.08	
2000–2499		1.03	1.00-1.06	
Gestational age (ref. GA ≥ 37)				
≤ 31		1.08	0.64–1.82	
32–36		1.03	0.87–1.21	

IRR: Incident rate ratio; CI: Confidence interval; BW: Birthweight; GA: Gestational age; all models were adjusted for sex, birth rank, mother’s education, mother’s age at childbirth, household socioeconomic status, and place of residence.

We did not observe a significant association between gestational age and school entry and age at school entry (Tables 3 and 4). Birthweight and gestational age were not associated with either repetition or dropout in our data (Table 3). The association between birthweight and school entry was stratified by sex, mother’s education, household socioeconomic status, and place of residence (Table 5). The association between birthweight and age at school entry was not stratified given the small sample size.

When examining the association between birthweight and school entry by stratifying by child sex, results reveal that, among boys, the probability of school entry decreased by 51% (HR: 0.49, 95% CI: 0.30–0.68) and 10% (HR: 0.90, 95% CI: 0.79–0.99) for children born weighing less than 2000 g and between 2000 and 2499 g, respectively (Table 5). Being born with a birthweight of less than 2000 g is associated with reduced chances of entering school for both children born to uneducated mothers and those born to educated mothers. In fact, children whose birthweight was less than 2000 g born to uneducated and educated mothers were 38% (HR: 0.62, 95% CI: 0.49–0.87) and 30% (HR: 0.70, 95% CI: 0.48–0.91) less likely to start school, respectively (Table 5). However, there is no statistical difference between these two groups of children (RHR: 1.03, 95% CI: 0.60–1.46) (Table 5). Even if birthweight between 2000 and 2499 g is detrimental to school entry only for children whose mothers are uneducated, there is no difference between these children and those whose mothers are educated (RHR: 1.03, 95% CI: 0.88–1.18). Stratified results by household socioeconomic status show that being born in a poor household with a birthweight less than 2000 g or between 2000 and 2499 g is associated with a decreased probability of school entry (Table 5). In less-poor households, birthweight is detrimental if it is less than 2000 g (HR: 0.71, 95% CI: 0.55–0.86). However, the effect of birthweight tends to be similar in poor and less-poor households. In formal and informal neighborhoods, a birthweight below 2000 g is associated with a significant reduction in the probability of entering school, with almost equivalent effects (Table 5).

**Table 5:** Association of birthweight with school entry across groups

Birthweight (ref. BW ≥ 2500)	Characteristics	HR(1)	95% CI	HR(2)	95% CI	RHR [(2)/(1)]	95% CI	
	Child’s sex	Boy		Girl				
≤ 1999		0.49	0.30–0.68	0.86	0.66–1.07	1.76	0.96–2.55	
2000–2499		0.90	0.79–0.99	0.93	0.85–1.02	1.04	0.89–1.19	
	Mother education	No education		Some education				
≤ 1999		0.68	0.49–0.87	0.70	0.48–0.91	1.03	0.60–1.46	
2000–2499		0.90	0.81–0.99	0.93	0.84–1.03	1.03	0.88–1.18	
	Socioeconomic status	Less-poor		Poor				
≤ 1999		0.71	0.55–0.86	0.63	0.31–0.95	0.90	0.40–1.39	
2000–2499		0.92	0.78–1.06	0.92	0.84–0.99	1.00	0.83–1.17	
	Place of residence	Formal area		Informal area				
≤ 1999		0.71	0.48–0.94	0.68	0.50–0.86	0.96	0.55–1.36	
2000–2499		0.91	0.79–1.02	0.92	0.84-1.00	1.01	0.86–1.17	

HR: Hazard rate; CI: Confidence interval; BW: Birthweight; RHR: Ratio of hazard rates; all models were adjusted for sex, birth rank, mother’s education, mother’s age at childbirth, household socioeconomic status, and place of residence.

## Discussion

This study provides new insight on children’s enrollment and their performance at school. We observed evidence of an association between birthweight and school entry. However, there was no evidence of association between birthweight and grade repetition or school dropout. There was no evidence of association between gestational age and school entry, grade repetition, or dropout. When they have the opportunity to enter school, low-birthweight children are more likely to have a delayed school entry (Table 4).

The stratified results show that whatever the household socioeconomic status, mother’s education level, and place of residence, the effect of birthweight on school entry remains similar. The results suggest that low birthweight creates deficits in children that are difficult to overcome, whatever household socioeconomic status, place of residence, and mother’s level of education. The probability of school entry is significantly decreased for boys born with a low birthweight. Points of estimates are different between boys and girls, but ratios of hazard rates are not statistically significant, which suggests that there is no difference between them. Again, we did not find any studies in the literature with which we could compare our results.

Two hypotheses can be evoked to explain the low probability of school entry and the delay of school entry of low-birthweight children. The first hypothesis is that low-birthweight children are more likely to have poor language, cognitive, and motor development compared to normal-birthweight children at the legal school entry age, which would result in non-enrollment or a delay of school entry. Studies conducted in Brazil and Haiti showed that low-birthweight children had poor cognitive performance at five years of age, lower expressive communication skills, and lower motor and cognitive development [44, 45]. At age seven, very low birthweight was associated with deficits in reading, poor cognitive development, and more behavioral problems compared to normal-birthweight children [46]. These conditions may lead parents to postpone or delay their child’s enrollment in school.

The second hypothesis is that, at school entry age, children born with a low birthweight are more likely to be shorter or thinner; therefore, parents might consider them “unready for school at the minimum age of enrollment” [47]. In a study conducted in the US state of Tennessee using data from infant and children born between 1975 and 1985, Binkin and colleagues [48] found that infants with lower birthweights were likely to remain shorter and lighter throughout childhood. A recent study undertaken in 32 countries in Sub-Saharan Africa revealed that low-birthweight children are more likely to be stunted, underweight, and wasted [49]. Similar results were found in other contexts [50, 51]. When children are shorter, they may have lower chances of starting school at the right moment. For example, Beasley and his colleagues revealed in their study in Tanzania that children were enrolled in school by teachers and parents on basis of height in absence of good estimates of age [52]. Children were enrolled in school or considered ready to start school when they were “able to reach their arm over the top of their head and touch the ear on the opposite side” [52]—a test that supposedly estimates their physical ability to make their way to school on foot. A test like this does not favor smaller, thinner children for school, as they judged unable to walk long distances [52]. This hypothesis would be more plausible if future research confirms our findings.

As stated, there was no association between school entry and gestational age. The lack of association may be explained by difficulties in measuring gestational age based on the last menstrual period in African settings where ultrasounds are rarely available [53]. If an association existed, we would not necessarily be able to identify it. Other studies unexposed to measurement bias could be conducted to more accurately determine the existence of an association between gestational age and school entry.

Like other studies, we could not find an association between birthweight or gestational age and academic performance outcomes such as grade repetition and dropout. For example, a study undertaken in a population of Chinese children shows that birthweight was not associated with years of schooling, college completion, and school performance in adolescence [21]. Another study conducted in Sweden using sibling data suggested that there was no association between gestational age and school performance [54].

In our study, the lack of association of birthweight or gestational age with grade repetition and dropout may be explained by other factors. First, this study has been conducted in Ouagadougou, capital city of Burkina Faso, which is better served by health centers and school infrastructure than the rest of the country. Cities generally have significant modern health care system [55]. Urban populations from both formal and informal areas have better access to health care [56] and they generally go to a health center when their children are sick [24]. Urban populations also have the advantage of benefiting from public health interventions, including maternal and child health programs [55]. These urban health advantages can significantly reduce the effects of development at birth on school performance. The results could, therefore, be different in a rural context. Second, the study focuses on the early stages of schooling; it could be that the effect of birthweight on school performance is more marked later in school.

### Strengths and limitations

This study’s key strengths include its prospective longitudinal design. Data allow researchers to follow children into their early school years and to measure their school trajectory. To our knowledge, this is the first study to analyze school entry, grade repetition, and school dropout simultaneously. It also has the advantage of exploring the modifying effects of household socioeconomic status and place of residence.

The study also has some limitations, however. Data on gestational age are subject to non-differential misclassification bias due to misreporting of the start date of the pregnancy, which may result in an underestimation of the association between gestational age and school trajectory. Another limitation of this study is the low number of children in the very low birthweight and very preterm categories, and lack of statistical power evidenced by the wide confidence intervals of our estimates. Also, the fact that some children in our sample did not enroll in school may be a source of selection bias for the analysis of grade repetition and school dropout. To overcome this limitation, data were weighted by the inverse probability weighting of selection. Finally, our results are representative of the area studied by the OHDSS and cannot be generalized to the entire city of Ouagadougou or any other African cities. Nonetheless, the study provides insight into what can be expected in similar settings.

## Conclusion

To our knowledge, this is the first study to assess the association between child development at birth and the different dimensions of school trajectory (school entry, age at school entry, grade repetition, and dropout). Results from this study show that low birthweight is associated with low probability of school entry. This probability tends to be comparable whatever the mother’s education level, household socioeconomic status, and place of residence. Low birthweight is also associated with a delayed school entry—that is, low-birthweight children start school at a later age. These results suggest that efforts to avoid low birthweights should be part of maternal and prenatal health care because the associated difficulties may be difficult to overcome later in the child’s life. Birthweight was not associated with grade repetition or dropout, however. Likewise, there was no evidence of an association between gestational age and school trajectory. Further longitudinal studies are needed to better understand the relationship between development at birth and school trajectory.

## Data Availability

The data that support the findings of this study are available from the Institut Superieur des Sciences de la Population (ISSP, http://www.issp.bf/index.php/fr/) but restrictions apply to the availability of these data, which were used under license for the current study, and so are not publicly available. Data are however available from the corresponding author upon reasonable request and with permission of the Institut Superieur des Sciences de la Population, which can be contacted at the following address: directeur@issp.bf.

## Abbreviations

OHDSS: Ouagadougou health and demographic surveillance system
FIML: Full information maximum likelihood
IPW: Inverse probability weighting
sd: Standard deviation
HR: Hazard rate
CI: Confidence interval
BW: Birthweight
GA: Gestational age
RHR: Ratio of hazard rates
IRR: Incidence rate ratio
